# Autophagy-Mediated Synaptic Refinement and Auditory Neural Pruning Contribute to Ribbon Synaptic Maturity in the Developing Cochlea

**DOI:** 10.3389/fnmol.2022.850035

**Published:** 2022-03-04

**Authors:** Rui Guo, Yice Xu, Wei Xiong, Wei Wei, Yue Qi, Zhengde Du, Shusheng Gong, Zezhang Tao, Ke Liu

**Affiliations:** ^1^Department of Otolaryngology Head and Neck Surgery, Beijing Friendship Hospital, Capital Medical University, Beijing, China; ^2^Department of Otolaryngology Head and Neck Surgery, Xiaogan Central Hospital, Wuhan University of Science and Technology, Xiaogan, China; ^3^Department of Otology, Shengjing Hospital, China Medical University, Shenyang, China; ^4^Department of Otolaryngology Head and Neck Surgery, Renmin Hospital of Wuhan University, Wuhan, China

**Keywords:** auditory development, cochlear ribbon synapses, autophagy, synaptic refinement, neural fibers pruning

## Abstract

In rodents, massive initial synapses are formed in the auditory peripheral nervous system at the early postnatal stage, and one of the major phenomena is that the number of afferent synapses in the cochlea is significantly reduced in the duration of development. This raises the hypothesis that the number of cochlear ribbon synapses are dramatically changed with hearing development and maturation. In this study, several tracers identifying activities of autophagy were applied to estimate the level of autophagy activity in the process of ribbon synapse development in mice; further, changes in the synaptic number and spiral ganglion nerve (SGN) fibers were quantitatively measured. We found robust expression of LC3B and lysosomal-associated membrane protein 1 as well as LysoTracker in or near inner hair cells and cochlear ribbon synapses in the early stage of postnatal development. Moreover, we found a significant loss in the intensity of SGN fibers at ribbon synaptic development and hearing onset. Thus, this study demonstrates that ribbon synaptic refinement and SGN fibers pruning are closely associated with the morphological and functional maturation of ribbon synapses and that synaptic refinement and SGN fiber pruning are regulated by the robust activities of autophagy in the earlier stages of auditory development.

## Introduction

Acoustic signals are sensed by cochlear hair cells and transmit information to the central nervous system *via* the spiral ganglion neurons (SGNs) (Liu et al., 2019; Wei et al., 2021). In mammals, this task is accomplished by using several synaptic structures with special features along the hearing pathway (Raphael and Altschuler, 2003; Wichmann and Moser, 2015). Among these synapses, ribbon synapses are the first synaptic structures formed between inner hair cells (IHCs) and SGNs, which have their own morphological and electrophysiological properties, ensuring rapid, accurate, and reliable signal transmission from the ear to the brain (Fuchs et al., 2003; Wichmann and Moser, 2015). Electron microscopy evidence has shown that a lack of synaptic ribbons can cause a reduction in the compound action potential and sound-evoked firing rates of SGNs (Jean et al., 2018).

Mice are born without hearing and begin to develop auditory capacity approximately 2 weeks after birth (Ehret, 1985; Safieddine et al., 2012). In the developing cochlea, ribbon synapses undergo robust changes both in morphology and function. During the early stage of postnatal development, excess synapses are formed, which are subsequently significantly eliminated to achieve optimal synaptic connectivity (Sobkowicz et al., 1982; Roux et al., 2009; Huang et al., 2012). Furthermore, studies have reported that lysosomal and autophagy activities are remarkably involved and contribute to the pruning of neural fibers (He et al., 2019), and synaptic elimination is a secondary change after axon or fiber pruning (Rowland et al., 2006; Song et al., 2008; Shen and Ganetzky, 2009; Ban et al., 2013; Chen et al., 2013; Xiong et al., 2020a), providing an insight into the possible mechanisms underlying the development and maturation of ribbon synapses in the developing cochlea. Consistently, the ribbon synapses in the developing cochlea were also found to undergo refinement coupled with exocytosis and Ca^2+^ influx (Moser and Beutner, 2000; Beutner and Moser, 2001; Johnson et al., 2005; Beurg et al., 2010; Wong et al., 2014). Our previous study showed that autophagy is required for the remodeling of cochlear ribbon synapses in postnatal mice (Xiong et al., 2020a). Despite this evidence, however, synaptic refinement and SGN fiber pruning have not yet been fully explored; moreover, it is still unclear whether synaptic refinement and SGN fiber pruning are fully mediated *via* dynamic autophagy flux (Ding et al., 2020; Zhou et al., 2020; Fu et al., 2021; Guo et al., 2021). Thus, in this study, we applied multiple markers to trace autophagy flux coupled with synaptic changes in the developing cochlea of mice, and hearing and synaptic function were estimated *via* auditory brainstem response (ABR) threshold detection and wave I amplitude analysis. Our study showed that quantitative reduction and functional facilitation of ribbon synapses, as well as density loss of SGN fibers, are regulated by dynamic autophagy flux, suggesting that both ribbon synaptic refinement and auditory neural fiber pruning are regulated *via* the dynamic activities of autophagy, which plays a key role in the development and construction of cochlear ribbon synapses.

## Materials and Methods

### Animals

Postnatal male C57BL/6J mice with documented at the age of P1, P7, P14, and P28 were purchased from Vital River Laboratory Animal Technology, Beijing, China. A total of 60 animals were used in the experiment. All animal experiments were approved by the Animal Ethics Committee of Capital Medical University. And all the efforts were aimed to minimize animals’ suffering and the number of mice which was sacrificed in the experiment.

### Drug Administration

3-MA (Millipore, 3089588) was dissolved in 0.9% saline as a stock solution (30 mg/ml) and stored at −20°C. The solution was heated to 60°C to completely dissolved and then cool at room temperature before being used. Experimental mice received intraperitoneal injections of 3-MA at a dose of 30 mg/kg daily from P7 to P14 consecutively, the controls were administered by the same amount of saline.

### Auditory Brainstem Responses

Auditory brainstem responses detected auditory function of mice at P1, P7, P14, and P28, respectively. All animals were anesthetized *via* intraperitoneal injection of ketamine (100 mg/kg, Gutian Pharmaceutical Co., Ltd., Fujian, China) plus xylazine (10 mg/kg, Sigma-Aldrich Co., Llc., United States). Needle electrodes were placed subcutaneously beneath the pinna of the test ear (−) and at the vertex (+), with a ground electrode placed in the contralateral ear over neck muscles. ABR threshold was recorded in a double-walled, electrically shielded, and radio frequency-shielded sound booth. ABR stimulus frequencies of 4, 8, 16, and 32 kHz and clicks (100 μs) were tested with System 3 hardware (Tucker Davis Technologies, Alachua, FL, United States) and SigGen/BioSig software (Tucker Davis Technologies). The stimulus level was calibrated and a probe tube microphone was tightly fitted into the external auditory canal. The ABR threshold was obtained for each animal by reducing the stimulus intensity in 10 dB steps and then 5 dB steps to identify the lowest intensity eliciting a response. The ABR threshold was defined as the lowest stimulus intensity that produced reliable and reproducible (in at least two trials) ABR waves. The amplitude of the wave I was identified as the difference between the first peak in the waveform and the baseline.

### Immunofluorescence

Mice were sacrificed after anesthesia with xylazine and ketamine (ketamine, 100 mg/kg and xylazine, 10 mg/kg). The acidotropic agent LysoTracker Red DND-99 (LysoT; Molecular Probes) was freshly diluted in growth medium to a final concentration of 100 nM. Cochlear tissues were dissected and preincubated with LysoT for 30 min at 37°C. And the tissues were subsequently harvested and fixed in 4% paraformaldehyde for 1h at room temperature. After fixation, tissues were permeabilized in 30% sucrose for 20 min, blocked in 0.3% TritonX-100 (Sigma, United States) for 30 min and 10% normal goat serum (ZSGB-BIO, China) for 1 h, then incubated overnight at 4°C with primary antibodies including: mouse anti-CtBP2 (1:500, Abcam, ab204663), rabbit anti-LC3B (1:100, CST 3868T), rat anti-lamp1 (1:300, Abcam, ab25245), chicken anti-NF200 (neurofilament 200) antibody (1:600, Chemicon, AB5539). The following day the preparations were washed three times in PBS for 5 min each and incubated with species-appropriate secondary antibodies. All the secondary antibodies were conjugated with Alexa Fluor TM 488, 568 (1:300, Invitrogen/Molecular Probes, Carlsbad, CA, United States catalog number: A21131, A21124). Specimens were subsequently washed three times in PBS and then mounted on glass slides using fluorescent mounting (ZSGB-BIO, ZLI-9557).

### Confocal Microscope Imaging

Images were acquired with a 63 oil-immersion, high-resolution confocal microscope (TCS SP8 II; Leica Microsystems, Wetzlar, Germany). Scanning was performed from top to bottom with an interval of 0.35 μm/layer, and images were then superimposed. Specimens were observed using optimal excitation wavelengths of 488 nm (green) and 568 nm (red). DAPI was observed using an optimal excitation wavelength of 358 nm (blue).

### Calculation of the Number and Size of Ribbon Synapses

Quantification of LC3B puncta, LAMP1 puncta, LysoT puncta, auditory nerve fibers (ANF) (labeled by anti-NF200) and ribbon synapses (labeled by anti-CtBP2) was performed at P1, P7, P14, and P28, respectively. In addition, ribbon synapses were quantified in the cochlear apex turn, middle turn and basal turn. We selected five samples in each group to calculate the average number of fluorescent puncta per IHC. The areas of presynaptic ribbons were measured in Adobe Photoshop CS6 software by segmenting the synaptic elements from the whole image into a single area then the area of the synapse was obtained by the measurement area tool.

### Measurement of the Distance Between Ribbon Synaptic Spots and Nucleus of Inner Hair Cell

The distance from ribbon synapses to nucleus center of IHCs was measured by using the scale tool in Adobe Photoshop CS6 software. In this study, the distance has been defined as the length from the synaptic immunostained spot to the nearest IHC. The actual distance was obtained by conversion with the image scale.

### Statistical Analysis

Statistical analysis was performed using GraphPad Prism 8 software (GraphPad Software Inc., La Jolla, CA, United States). Normally distributed continuous variables were presented as means ± standard error of the mean (SEM). Statistical differences between groups in ABR threshold, ABR wave I amplitude, fluorescent puncta (LC3B, LAMP1, LysoT, NF200, and ribbon synapses) per IHC were analyzed using two-way analysis of variance (ANOVA), followed by Bonferroni’s multiple comparisons test. *p < 0.05, **p < 0.01, ***p < 0.001, and ****p < 0.0001.

## Results

### Auditory Detection in the Developing Cochlea of Mice

**FIGURE 1:**
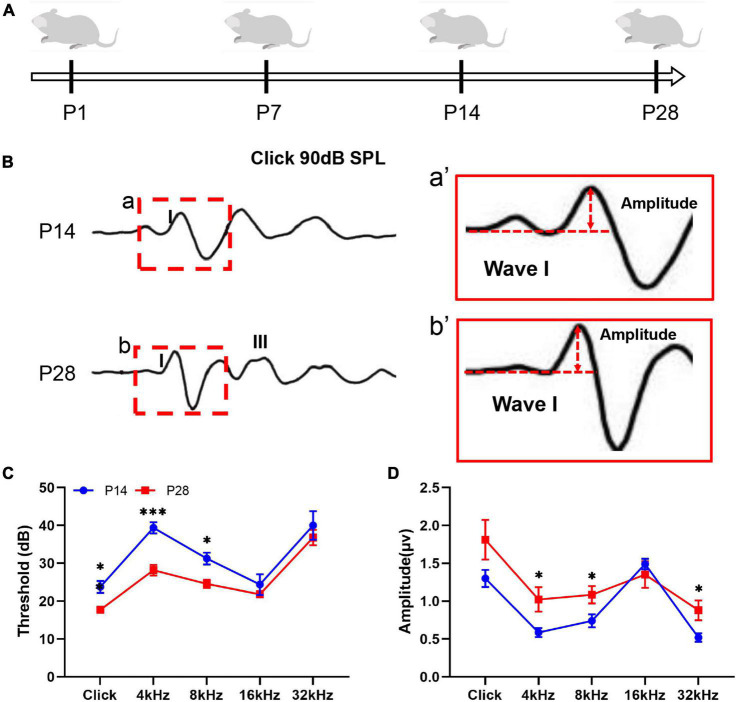
Hearing detection corresponding to developing cochlea. **(A)** Schematic diagram of the experimental procedure and time points for hearing detection. Hearing function in the mice was estimated by detecting the ABR threshold and ABR wave I amplitude at P14 and P28. **(B,a,b)** A visible ABR waveform was observed at P14 and P28; the red dashed frame indicates ABR wave I. **(a′,b′)** A larger amplitude of ABR wave I was found at P28 than at P14. **(C)** Compared with P14, significantly reduced ABR thresholds at P28 were identified at click, 4 kHz, and 8 kHz (*p < 0.05; ***p < 0.001). **(D)** Compared with P14, significantly increased ABR wave I amplitudes were observed at P28 at 4, 8, and 32 kHz (*p < 0.05). ABR, auditory brainstem response.

To investigate the hearing development profile, we first detected changes in the ABR threshold and amplitude of ABR wave I (Figures 1A–D). In this study, a visible ABR waveform was first recorded at P14, and a clearer ABR waveform appeared at P28 (Figures 1A,B). Because the amplitude of the ABR wave I has been demonstrated to roughly reflect the function of cochlear ribbon synapses (Qi et al., 2019; Xiong et al., 2020b), we then estimated the changes in the amplitude of the ABR wave I and found a significantly increased amplitude of the ABR wave I at P28 at 4, 8, and 32 kHz compared with that at P14 (Figures 1a′,b′,D). Thus, our study suggested a gradual change in hearing construction coupled with ribbon synaptic maturity in the developing cochlea of mice.

### Changes in the Number of Ribbon Synapses in the Developing Cochlea

**FIGURE 2:**
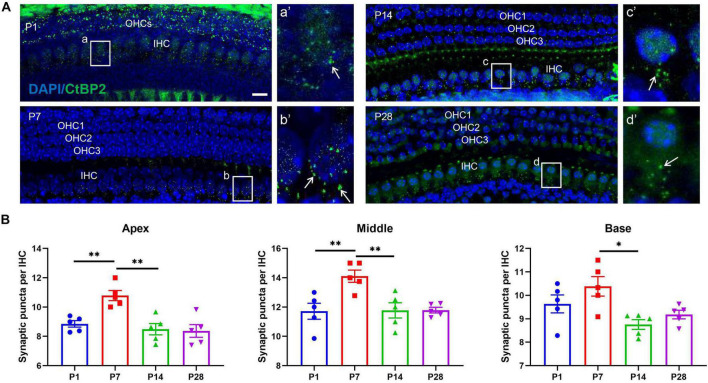
Alterations in the number of ribbon synapses in the developing cochlea. **(A)** Whole mount of immunostaining at P1, P7, P14, and P28. Ribbon synapses were identified beneath the nuclei of IHCs using anti-CtBP2/RIBEYE (green, white arrow), and the nuclei of IHCs and OHCs were identified using DAPI staining (blue). A number of synaptic spots can be seen at P1 [**(a)** white frame indicated; **(a′)** enlarged image of panel **(a)**], and the highest number of synaptic puncta was identified at P7 [**(b)**, white frame indicated; **(b′)** enlarged image of panel **(b)**]. In contrast, reduced puncta of ribbon synapses appeared at P14 [**(c)**, white frame indicated; **(c′)** enlarged image of panel **(c)**] and P28 [**(d)** white frame indicated; **(d′)** enlarged image of panel **(d)**]. Scale bar = 5 μm. **(B)** Quantitative analysis of changes in ribbon synaptic numbers in developing cochlea. Correspondingly, the largest number of ribbon synaptic puncta was found at P7 across the cochlear frequency (including the apex, middle, and basal turns), and a significantly reduced number of synaptic spots was observed at P14 and P28 (*p < 0.05; **p < 0.01). IHC, inner hair cell; OHC, outer hair cell.

First, we measured quantitative changes in the cochlear ribbon synapses in the developing cochlea. In this study, cochlear ribbon synapses were labeled with anti-CtBP2 (Liu et al., 2009; Wang and Green, 2011; Wong et al., 2014). Before onset of hearing, at P1, the immunostaining positive puncta of CtBP2/RIBEYE were 8.85 ± 0.22 (apex), 11.71 ± 0.54 (middle), 9.64 ± 0.38 (base), respectively; At P7, the numbers were 10.78 ± 0.35 (apex), 14.11 ± 0.42 (middle), 10.38 ± 0.42 (base) respectively; At P14, the numbers were 8.49 ± 0.39 (apex), 11.78 ± 0.52 (middle), 8.76 ± 0.21 (base); And at P28, the numbers were 8.37 ± 0.43 (apex), 11.79 ± 0.19 (middle), 9.18 ± 0.18 (base), respectively (Figure 2). In this study, the largest number of synaptic puncta appeared at P7 and was significantly reduced at P14 and P28, suggesting that the number of ribbon synapses may undergo robust synaptic elimination during cochlear development.

### Changes in the Sizes of Ribbon Synapses and the Distances From the Synapses to the Nuclei of Inner Hair Cells

**FIGURE 3:**
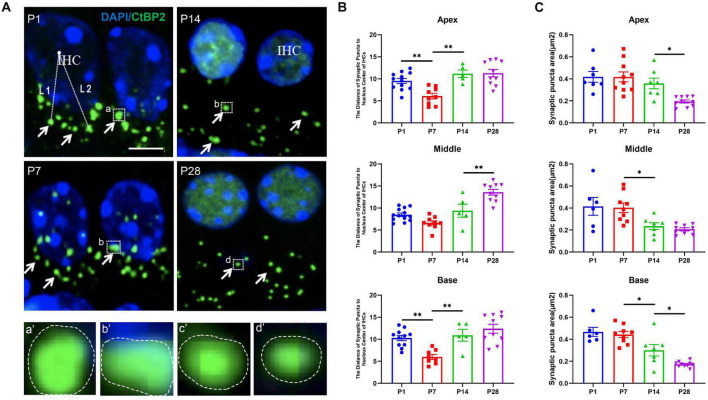
Calculation of the size of synaptic spots and the distance between synaptic puncta and the nucleic center in adjacent IHCs. **(A)** DAPI staining of the nuclei of IHCs (blue) and ribbon synaptic puncta was performed using anti-CtBP2 (green, white indicated). The white dashed line indicates the distance from the synaptic puncta to the nucleic center of the adjacent IHC (L1, L2); **(a–d)** represent different sizes of synaptic puncta at P1, P7, P14, and P28 (white dashed frame); **(a′–d′)** are enlarged images of panels **(a–d)**, respectively. Scale bar = 5 μm. **(B)** Quantitative analysis of the distance between synaptic puncta and the nucleic center of adjacent IHCs. Compared with P1, a significantly increased distance was observed at P14 and P28 (**p < 0.01, apical and basal), and a more significantly enhanced distance appeared at P28 (**p < 0.01, middle). **(C)** Quantitative analysis of synaptic spot size. The sizes of the synaptic puncta at P1 and P7 were significantly larger than those at P14 and P28 (*p < 0.05, middle and basal); at the apex, the sizes at P1, P7, and P14 were significantly larger than those at P28 (*p < 0.05, apex). IHC, inner hair cell.

To explore whether ribbon synaptic size undergoes significant alterations, we next estimated the changes in ribbon synaptic size at P1, P7, P14, and P28 using Adobe Photoshop CS6 software (San Jose, CA, United States). At P1, the mean sizes of synaptic spot were 0.42 ± 0.05 (apex), 0.42 ± 0.08 (middle), 0.47 ± 0.04 (base), respectively; At P7, the mean sizes were 0.42 ± 0.04 (apex), 0.40 ± 0.04 (middle), 0.44 ± 0.03 (base) respectively; At P14, the sizes were 0.36 ± 0.05 (apex), 0.24 ± 0.03 (middle), 0.30 ± 0.05 (base); And at P28, the sizes were 0.19 ± 0.01 (apex), 0.21 ± 0.01 (middle), 0.17 ± 0.01 (base), respectively; The largest synaptic spots appeared at P1 and P7, and a significant reduction in synaptic size was found at P14 and P28, suggesting that the size of synaptic puncta undergoes a similar refinement in the developing cochlea (Figure 3). Next, we estimated the changes in the distance from the synaptic puncta to the nuclei of the IHCs. At P1, the distances from the synaptic puncta to nuclei center of adjacent IHCs were 9.53 ± 0.58 μm (apex), 8.48 ± 0.39 μm (middle), 10.29 ± 0.60um (base); At P7, the distances were 6.09 ± 0.65 μm (apex), 6.63 ± 0.48 μm (middle), 5.98 ± 0.57 μm (base), respectively; At P14, the distances were 11.15 ± 0.84 μm (apex), 9.39 ± 1.48 μm (middle), 10.91 ± 1.34 μm (base); At P28, the distances were 11.30 ± 0.86 μm (apex), 13.59 ± 0.66 μm (middle), 12.39 ± 1.02 μm (base), respectively. The distance at the P7 apex and middle turn was significantly shorter than that at P14 and P28 (**p < 0.01), and the distance at the P14 basal turn was also significantly shorter than that at P28 (**p < 0.01) (Figure 3), suggesting a remarkable retraction of the postsynaptic neural fibers during ribbon synaptic maturation.

### LC3B Detection in the Region of Ribbon Synapses in the Developing Cochlea

**FIGURE 4:**
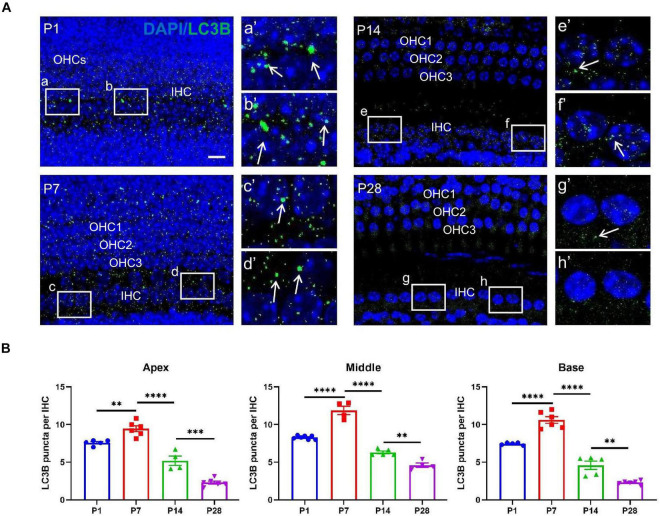
LC3B detection in the ribbon synapses regions. **(A)** Autophagic flux was identified using anti-CtBP2 (green, white arrows). White frames at P1 **(a,b)**, P7 **(c,d)**, P14 **(e,f)**, and P28 **(g,h)** indicate the regions of ribbon synapses; **(a′**–**h′)** are enlarged images of panels **(a–h)**, respectively. Scale bar = 5 μm. **(B)** Quantitative analysis of the number of LC3B puncta at P1, P7, P14, and P28. The highest number of LC3B puncta was identified at P7, and significant reductions in LC3B puncta were observed at P14 and P28 (*p < 0.01; ***p < 0.001; ****p < 0.0001).

To investigate whether autophagy flux undergoes remarkable alterations during cochlear development, we measured the level of LC3B, the current major recognized marker of autolysosomes (Barth et al., 2010; Aburto et al., 2012; Yuan et al., 2015; He et al., 2020, 2021; Liu et al., 2021), at P1, P7, P14, and P28, respectively. At P1, the number of positive immunostaining LC3B puncta were 7.59 ± 0.16 (apex), 8.30 ± 0.09 (middle), 7.45 ± 0.08 (base); At P7, the numbers were 9.44 ± 0.38 (apex), 11.88 ± 0.56 (middle), 10.60 ± 0.44 (base), respectively; At P14, the numbers were 5.21 ± 0.62 (apex), 6.30 ± 0.19 (middle), 4.60 ± 0.55 (base); At P28, the numbers were 2.31 ± 0.20 (apex), 4.60 ± 0.29 (middle), 2.31 ± 0.11 (base), respectively (Figures 4A,B). The highest number of LC3B puncta was observed at P7, suggesting the highest level of autophagic activity. Our study also showed decreased autophagy flux after P7 as a significantly reduced number of LC3B puncta were found at P14 and P28, and the smallest number of LC3B spots appeared at P28, suggesting that the level of autophagy was dramatically reduced when the cochlea was developed (Figures 4A,B).

### Lamp1 Detection in the Developing Cochlea

**FIGURE 5:**
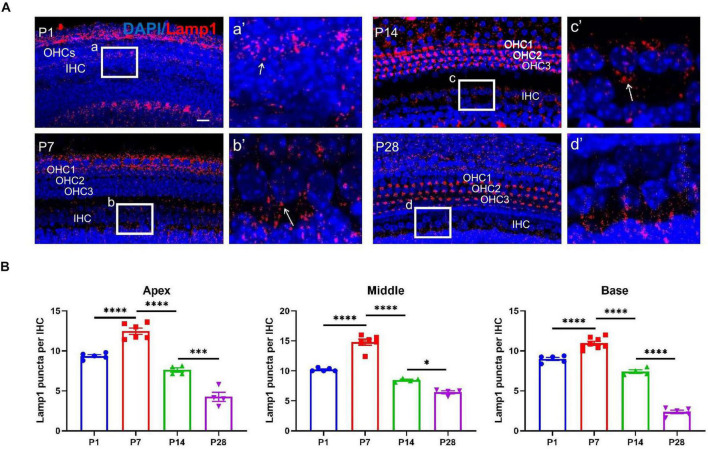
LAMP1 detection in the ribbon synapses regions. **(A)** LAMP1 was identified using anti-LAMP1 (red, indicated), and white frames at P1 **(a)**, P7 **(b)**, P14 **(c),** and P28 **(d)** indicate the regions of ribbon synapses; **(a′–d′)** are enlarged images of panels **(a–d)**, respectively; white arrows indicate LAMP1-stained positive spots. Scale bar = 5 μm. **(B)** Quantitative analysis of the number of positive puncta on P1, P7, P14, and P28. Correspondingly, the highest number of LAMP1 puncta appeared at P7, and a remarkable loss of LAMP1 puncta was found at P14 and P28 across frequencies (apex, middle and base) (*p < 0.05; ***p < 0.001; ****p < 0.0001). LAMP1, lysosomal-associated membrane protein1.

To further confirm the autophagic activities in the developing cochlea, we examined additional autophagy-associated markers, such as lysosomal-associated membrane protein1 (LAMP1). LAMP1 has been used as a marker of autophagic flux (Yuan et al., 2015). At P1, the number of Lamp1 staining spots were 9.36 ± 0.17 (apex), 10.20 ± 0.13 (middle), 8.98 ± 0.20 (base); At P7, the numbers were 12.47 ± 0.40 (apex), 14.80 ± 0.52 (middle), 10.96 ± 0.24 (base), respectively; At P14, the numbers were 7.60 ± 0.27 (apex), 8.45 ± 0.14 (middle), 7.41 ± 0.22 (base); At P28, the numbers were 4.25 ± 0.57 (apex), 6.45 ± 0.25 (middle), 2.37 ± 0.22 (base), respectively. Consistent with LC3B detection, the highest level of LAMP1 at or near the IHC region was found at P7, and the level decreased gradually at P14 and P28 (Figures 5A,B).

### Detection of LysoTracker Red and Examination of Co-localization Between Ribbon Synapses and Auditory Nerve Fibers

LysoTracker Red dye, a type of lysosome marker, is used as a probe to track the formation of autolysosome-like structures and to indicate autophagic activity in the living tissues of various organisms by labeling acidic organelles such as autolysosomes (Aburto et al., 2012). Moreover, LysoTracker Red has been used to identify lysosomal activity, with positive staining indicating neuronal pruning (Song et al., 2008; He et al., 2021).

**FIGURE 6:**
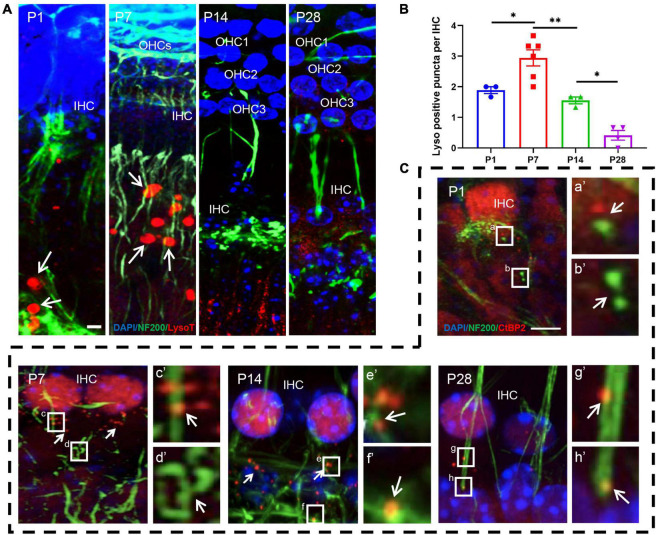
LysoTracker Red detection and examination of co-localization between ribbon synapses and auditory nerve fibers. **(A)** The signals of LysoTracker Red (red signals in round shape) were clearly observed at P1 and P7 (white arrows indicated); auditory nerve fibers were identified using anti-NF200 (green, corded). Signals for LysoTracker Red almost disappeared at P14 and P28. Scale bar = 5 μm. **(B)** Quantitative analysis of LysoTracker Red signals. The largest number of signals was found at P7, and a significant loss of LysoTracker Red signals was observed at P14 and P28 (*p < 0.05; **p < 0.01). **(C)** Poor co-localization between CtBP2-positive puncta and NF200-stained spots at P1: white frames **(a,b)** and enlarged images [**(a′,b′)** white arrows indicated] with the red and green signals clearly separated. A significantly improved co-localization level between red and green signals can be found at P7, P14 and P28; see c and d (P7), e and f (P14), g and h (P28), as well as their enlarged images [**(c′–h′)** respectively]. NF200, neurofilament 200.

Our study showed that lysosome-mediated auditory neuronal axon pruning was initially observed at P1, beginning with LysoTracker particles distributed at or near the bottom of the axon bulb (Figure 6A). At P7, the activity of lysosome-mediated axon pruning was significantly enhanced, and a large amount of LysoTracker was visible in the axon and neuronal fibers (Figures 6A,B). After the onset of hearing, LysoTracker staining almost vanished around the axon bulb between P14 and P28 (Figures 6A,B). These results suggest that robust lysosome-mediated auditory neuronal axon pruning occurred before the onset of hearing, and axon pruning was significantly decreased after hearing onset.

Next, we co-labeled CtBP2 and neurofilament 200 (NF200) using immunostaining. Only small amounts of NF200 puncta connected with the IHCs were observed at P1, and CtBP2-positive spots occurred in the vicinity of the IHCs. Most importantly, the two types of signals were clearly separated, and no merged signals could be found (Figures 6C,a,b,a′,b′). AtP7, excessive neuronal fibers and ribbon synapses were found to form, and merged signals increased significantly (Figures 6C,c,d,c′,d′). At P14 and P28, merged signals formed byanti-NF200- and anti-CtBP2-stained puncta increased robustly (Figures 6C,e–h,e′–h′). These results indicate that afferent neuron fibers undergo changes analogous to those of ribbon synapses.

### Changes in the Number of Auditory Nerve Fibers in the Developing Cochlea

**FIGURE 7:**
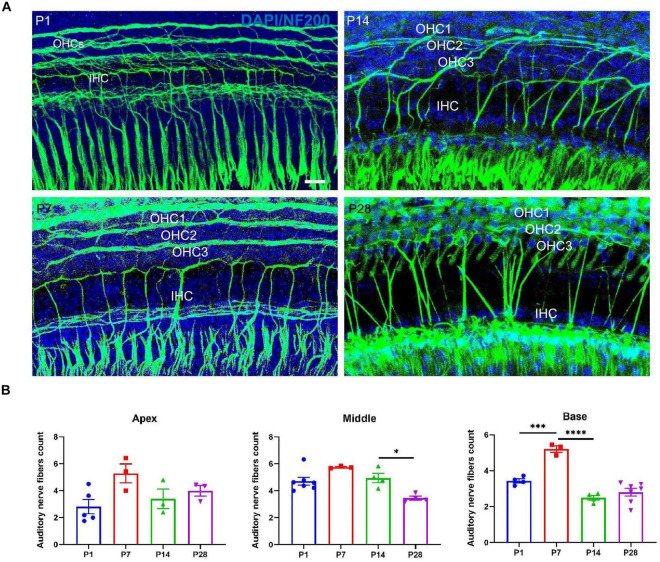
Examination of the number of auditory nerve fibers. **(A)** Auditory nerve fibers were traced using anti-NF200 (green), and a massive number of auditory nerve fibers were seen at P1 and P7; a reduced number of auditory nerve fibers appeared at P14 and P28. Scale bar = 5 μm. **(B)** Compared with P1 and P7, quantitative analysis showed a significant loss of auditory nerve fibers at P14 and P28 in the middle and basal turns (*p < 0.05) and no significant difference at the apical turn (*p* > 0.05), suggesting that postsynaptic auditory nerve fibers also undergo significant pruning in the developing cochlea (***p < 0.001; ****p < 0.0001). NF200, neurofilament 200.

To further investigate whether the postsynaptic auditory nerve fibers undergo corresponding changes in synaptic alterations, we detected alterations in the auditory nerve fibers. Auditory nerve fibers were traced using anti-NF200, and we observed a massive number of auditory nerve fibers at both P1 and P7; the number of auditory nerve fibers decreased significantly at P14 and P28 (Figure 7), suggesting that postsynaptic auditory nerve fibers also undergo remarkable pruning in developing cochlea.

### Inhibiting Autophagy Activity in the Developing Cochlea Hinders Synaptic Pruning and Impairs Hearing Function

**FIGURE 8:**
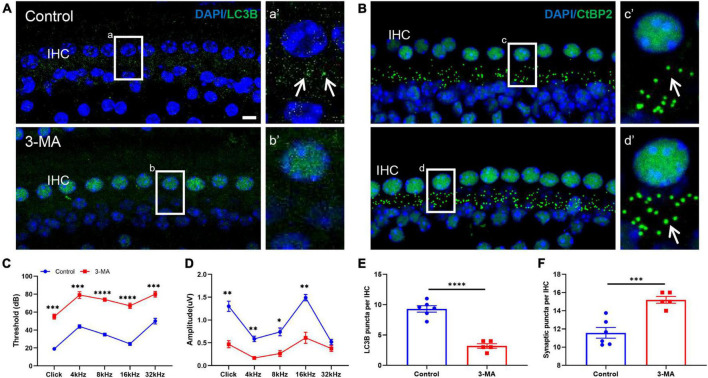
Inhibiting autophagic activity in the developing cochlea increased the number of ribbon synapses but impaired hearing function. **(A,a,b)** and their respective enlarged images **(a′,b′)** show that 3-MA administration can cause a significant loss of LC3B-positive puncta compared with the controls. **(B)** Normal CtBP2 puncta (green) can be seen in the control group; the white frame indicates **(c)**; **(c′)** is the enlarged image of panel **(c)**. 3-MA administration caused an increased number of CtBP2 puncta; the white frame indicates **(d)**, panel **(d′)** is the enlarged image of panel **(d)**. **(C)** ABR thresholds were significantly increased across frequencies in the 3-MA-administrated group compared with controls (***p < 0.001; ****p < 0.0001). **(D)** Compared with the controls, a significant reduction in ABR wave I amplitude appeared in the 3-MA group at click, 4, 8, 16, and 32 kHz (*p < 0.05; **p < 0.01). **(E)** A significant reduction in LC3B-positive puncta can be seen in the 3-MA group compared with the controls (****p < 0.0001). **(F)** A significant enhancement in the number of ribbon synaptic spots can be seen in the 3-MA group compared with controls (***p < 0.001). 3-MA, 3-methyladenine; ABR, auditory brainstem response.

In this study, we used 3-methyladenine (3-MA), an inhibitor of autophagy flux (Rubinsztein et al., 2007; Aburto et al., 2012; Liu et al., 2021), to investigate whether inhibiting autophagy activities in the developing cochlea significantly affects ribbon synaptic pruning. Here, 3-MA administration was applied at P7 and suspended at P14; interestingly, we found that LC3B-stained spots in IHCs nearly vanished compared with in the controls (Figures 8A,E). Next, we detected changes inCtBP2-positive spots; correspondingly, the number of CtBP2-positive spots in the 3-MA-treated group increased remarkably compared with in the normal controls (Figures 8B,F). Furthermore, we explored whether the inhibition of autophagy activities in the developing cochlea can impair hearing function. We estimated the ABR threshold and changes in the amplitude of ABR wave I and found a significantly enhanced ABR threshold, as well as a reduced amplitude of ABR wave I, suggesting that inhibiting autophagy activities in the developing cochlea impairs hearing function (Figures 8C,D).

## Discussion

Our findings showed that there may be a dramatic loss in the number of cochlear ribbon synapses during auditory development and maturation. Moreover, there is similar pruning of postsynaptic auditory nerve fibers during ribbon synaptic development and hearing onset. Finally, our findings demonstrated that robust autophagic activities in the early stage of auditory development regulate synaptic refinement and pruning of auditory nerve fibers.

The transformation of ribbon synapses during development is required for correct acoustic formation (Raphael and Altschuler, 2003). Thus, the ribbon synapses in the cochlea should undergo dramatic changes before the onset of hearing to meet the requirement of auditory development (Huang et al., 2007; Kros, 2007; Johnson et al., 2009; Roux et al., 2009). Our studies revealed the timeline of ribbon synapses during development and maturation, as well as the possible mechanism of synaptic elimination and neural pruning. In consistent with quantitative analysis of the synaptic number, the analysis of the size or shape of synaptic spot is also an indicator of synaptic plasticity. A previous study reported that ribbon synapse puncta are T-shaped or table-shaped at P1 (Vollrath and Spiwoks-Becker, 1996). Our results were partially different from those of a previous study because we found that the size of CtBP2 puncta in postnatal mice is larger than that of mature mice. Huang reported that CtBP2-positive puncta increased significantly in the first week of postnatal life (Huang et al., 2012), consistent with the data observed in this study. In our study, the maximum number of pre-synaptic CtBP2-positive puncta appeared at P7. Sending reported that the population of ribbon synapses is dynamic, and the synaptic structure gradually matures overtime (Sendin et al., 2007). Our data revealed consistent evidence that immature ribbon synapses with no function are excessively formed during the early stages of development. However, the mechanisms underlying the quantitative changes in ribbon synapses during synaptic development have remained unclear (Sobkowicz et al., 1982; Friedman et al., 2000; Roux et al., 2009). In this study, we proposed novel findings that changes in the number of ribbon synapses are driven by synaptic elimination and axon pruning, which may be attributed to lysosome-mediated autophagy flux that activates the onset of hearing.

During development, massive contacts between IHCs and SGNs are eliminated because the afferent fibers are refined or retracted, leading to a significant reduction in synapses (Sobkowicz et al., 1982; Huang et al., 2012). In this study, we found that the synaptic puncta increased robustly in both IHCs and outer hair cells at P7; this fluctuating timeline for the quantification and distribution of ribbon synapses is consistent with the previous report (Liberman and Liberman, 2016). During the development of the cochlea, the populations of type II SGNs obviously decrease because of the apoptosis of the neurons (Barclay et al., 2011).

In mice, hearing onset occurs around P12-14, with a significant decrease in pre- and post-synaptic puncta during this period. Before hearing onset, there is a large number of round- or ovular-shaped immature cochlear ribbon synapses; further, they are tethered with a few vesicles (Wong et al., 2014). After the onset of hearing, the ribbon synapses begin to mature, the ribbon shape is elliptical or rod-like, and each ribbon is associated with a single afferent fiber. Such a structure may be essential for the release of neuron transmitters (Nicol and Walmsley, 2002; Yang et al., 2010). An additional experiment demonstrated that maturation of the ribbon synapse is also mediated by thyroid hormones, indicating that many factors are involved in the process of synaptic elimination (Rüsch et al., 1998; Sendin et al., 2007). First, our data show a new mechanism underlying ribbon synaptic elimination and axon pruning in the cochlea through the robust cellular activity of lysosome-mediated autophagy.

In this study, several markers identifying autophagy activity were used to estimate the level of autophagy in the developing cochlea. Among these markers, LC3B is the most widely used indicator of autophagic activity (Barth et al., 2010; He et al., 2017). In addition, LAMP1 and LysoTracker were also used to detect autophagy flux because these two markers can trace lysosome-mediated autophagy (Aburto et al., 2012; Yuan et al., 2015). Application of the three markers can provide convincing evidence of autophagy activity, and in our study, all three markers traced similar autophagy flux in the developing cochlea, which strongly illustrates the refinement of ribbon synapses and the pruning of postsynaptic neurofilaments during cochlear development. Furthermore, treatment with 3-MA, an inhibitor of autophagic flux (Rubinsztein et al., 2007; Aburto et al., 2012), caused a significant increase in the number of ribbon synapses, demonstrating that this synaptic effect is mediated *via* autophagy in the cochlea. In this study, we found a significant reduction of auditory neural fibers at P14&28 in response to the synaptic loss, we hypothesis that it could be due to the decreased number of synaptic signals, so that postsynaptic auditory nerve fibers are unable to find sufficient targets to built up synaptic contacts. Previous study has proposed that the level of autophagic activity in the cochlea of maturated mice decreases significantly and is nearly undetectable (Xiong et al., 2020a), in this study, we have found consistent results (data not shown), suggesting that autophagy is a critical regulator in ribbon synapse maturation in developing cochlea.

Our study found a relatively stable number of ribbon synapses at P28. The stability of ribbon synapses could indicate a strengthening of the synapses to meet the increasing need for sound coding; the data is consistent with previous reports (Shi et al., 2015; Yu et al., 2015). However, there is a lack of functional evidence in this study; for example, although we estimated the hearing function of the mice, we did not detect functional alterations in the level of IHCs corresponding to the activity of lysosome-mediated autophagy. With the utilization of combined approaches, including high-resolution optical imaging, electrophysiological recording, and patch-clamp electrophysiology, more detailed information on the mechanisms will be revealed in the future.

## Data Availability Statement

The original contributions presented in the study are included in the article/supplementary material, further inquiries can be directed to the corresponding author/s.

## Ethics Statement

The animal study was reviewed and approved by Animal Ethics Committee of Capital Medical University.

## Author Contributions

KL contributed to the design of the study, analyzed and interpreted the result. SG and ZT contributed to the design of the study. RG, YX, WX, WW, YQ, and ZD conducted the experiments. RG, YX, WX, SG, and KL analyzed the generated data. RG, YX, and KL wrote the manuscript. All authors reviewed and approved the final version of the manuscript.

## Conflict of Interest

The authors declare that the research was conducted in the absence of any commercial or financial relationships that could be construed as a potential conflict of interest.

## Publisher’s Note

All claims expressed in this article are solely those of the authors and do not necessarily represent those of their affiliated organizations, or those of the publisher, the editors and the reviewers. Any product that may be evaluated in this article, or claim that may be made by its manufacturer, is not guaranteed or endorsed by the publisher.
